# A novel ex-vivo isolated rabbit heart preparation to explore the cardiac effects of cervical and cardiac vagus nerve stimulation

**DOI:** 10.1038/s41598-023-31135-4

**Published:** 2023-03-14

**Authors:** Bettina Kronsteiner, Max Haberbusch, Philipp Aigner, Anne-Margarethe Kramer, Patrick M. Pilz, Bruno K. Podesser, Attila Kiss, Francesco Moscato

**Affiliations:** 1 Center for Medical Physics and Biomedical Engineering, Medical University of Vienna, Vienna, Austria; 2 Ludwig Boltzmann Institute for Cardiovascular Research, Vienna, Austria; 3 Center for Biomedical Research, Medical University of Vienna, Vienna, Austria; 4 Austrian Cluster for Tissue Engineering, Vienna, Austria

**Keywords:** Biomedical engineering, Neuroscience, Medical research

## Abstract

The cardiac responses to vagus nerve stimulation (VNS) are still not fully understood, partly due to uncontrollable confounders in the in-vivo experimental condition. Therefore, an ex-vivo Langendorff-perfused rabbit heart with intact vagal innervation is proposed to study VNS in absence of cofounding anesthetic or autonomic influences. The feasibility to evoke chronotropic responses through electrical stimulation ex-vivo was studied in innervated isolated rabbit hearts (n = 6). The general nerve excitability was assessed through the ability to evoke a heart rate (HR) reduction of at least 5 bpm (physiological threshold). The excitability was quantified as the charge needed for a 10-bpm HR reduction. The results were compared to a series of in-vivo experiments rabbits (n = 5). In the ex-vivo isolated heart, the baseline HR was about 20 bpm lower than in-vivo (158 ± 11 bpm vs 181 ± 19 bpm). Overall, the nerve remained excitable for about 5 h ex-vivo. The charges required to reduce HR by 5 bpm were 9 ± 6 µC and 549 ± 370 µC, ex-vivo and in-vivo, respectively. The charges needed for a 10-bpm HR reduction, normalized to the physiological threshold were 1.78 ± 0.8 and 1.22 ± 0.1, in-vivo and ex-vivo, respectively. Overall, the viability of this ex-vivo model to study the acute cardiac effects of VNS was demonstrated.

## Introduction

There is growing evidence showing that various pathological conditions are associated with and affected by autonomic imbalances that manifest as dominance of the sympathetic over the parasympathetic activity^1–4^. Therefore, vagus nerve stimulation (VNS) has been considered to be a promising therapeutic approach to treat diverse pathological conditions, such as epilepsy^5–8^, depression^9^, and cardiac diseases^10–14^ by restoring the vagal tone to a physiological level.

In cardiac medicine, VNS has proven to promote cardioprotective and anti-fibrillatory effects, thus providing a promising therapeutic approach for non-pharmacological treatment of various cardiac pathological conditions, such as ventricular arrhythmias^15^, atrial fibrillation^13^ and heart failure^16,17^. However, the fact that VNS is mostly applied to the cervical level due to surgical ease of access and the possibility to target various organ-related fibers, it is often accompanied by difficultly controllable systemic off-target effects^18–20^.

Although numerous studies have explored diverse effects of VNS on HR and hemodynamic function, in-vivo^11,21–24^ and in-situ^10,25–27^, including anti-antiarrhythmic^15^ and cardioprotective effects^28^, or alleviation of hypertension^29^, the outcomes are diverse, and the impact of VNS on the cardiac activity is still not fully understood. One main hindrance to better understand the cardiac effects of VNS is the presence of autonomic reflexes in-vivo, anesthetic and analgesic effects, and inter-individual variations.

Addressing this problem, the purpose of this study was to establish a novel model of vagally innervated and fully isolated rabbit heart in order to study the cardiac effects of ex-vivo VNS under well-controllable und reproducible experimental conditions. The results were then compared to a series of in-vivo experiments.

## Methods

### Surgical preparation and instrumentation of the vagus nerve in-vivo and ex-vivo

#### Ethical approval

For all experiments, female rabbits (New Zealand White, n = 5 in-vivo and n = 6 ex-vivo, 2.5–3.3 kg body weight, age of 3–4 months) were used. All experiments were approved by the Institutional Animal Care and Use Committee of the city of Vienna (BMBWF 2020-0.016.858-GZ 2020-0.016.858) and conducted following relevant guidelines and regulations. Experiments were conducted and reported in accordance with the ARRIVE guidelines. All surgical procedures were carried out under deep anesthesia and analgesia in ventilated animals.

#### Anesthesia

Animals were premedicated using intramuscular injections of ketamine (Ketasol^®^, Richter Pharma, 50 mg/ml, 0.6 ml/kg bodyweight) and dexmedetomidine (Dexdomitor^®^, Zoetis, 0.5 mg/ml, 0.2 ml/kg bodyweight). Maintenance of anesthesia was achieved using sevoflurane (Sevorane^®^, AbbVie AG, Baar, Switzerland) dissolved in 4 l/min of 100% oxygen through an endotracheal tube (inner diameter 2.5 cm). Fentanyl (Fentanyl Hameln, 50 μg/ml, 0.2 ml/kg/h, Hameln pharmaceuticals GmbH, Hameln, Germany) was administered intravenously for analgesia. In addition, fluids and electrolytes were provided by crystalloid solution (Elo-Mel isoton, Fresenius Kabi, Graz, Austria) and lactate-buffered Ringer’s solution, respectively, to maintain physiological blood pH and electrolyte levels. Blood gas was regularly measured and kept within physiological ranges (pO_2_: 95–100 mmHg, pCO_2_: 35–45 mmHg, pH 7.35–7.45) by adjustment of the ventilation frequency (25-26/min) and of the tidal volume (10 ml/kg body weight).

#### Vagus nerve dissection in-vivo

**Figure 1:**
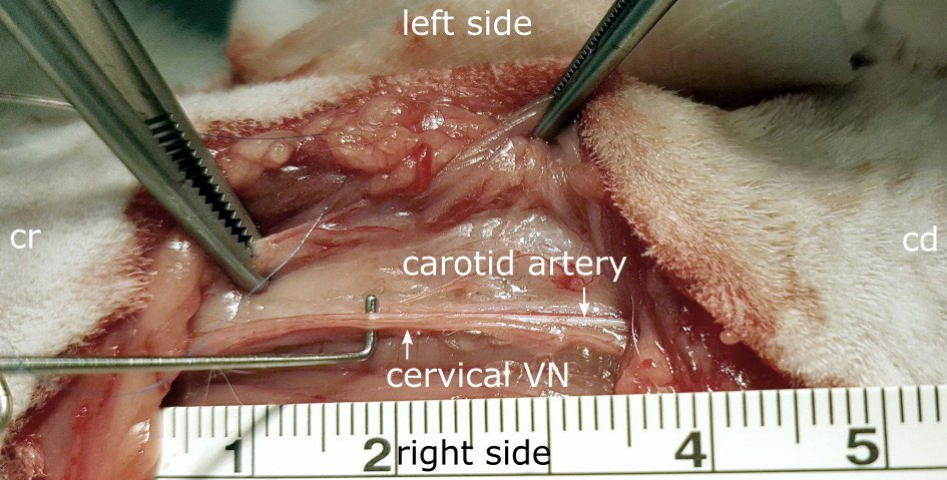
Surgical window showing the dissection of the right vagus nerve (VN) at the cervical level for instrumentation with cuff electrodes in a rabbit. The carotid artery runs parallelly to the cervical VN. *Cr* cranial, *cd* caudal.

For in-vivo, a surgical window of three to five cm was opened at the cervical level (Fig.1). The carotid sheath, containing the vagus nerve, the carotid artery, the internal jugular vein and the sympathetic trunk was opened. The cervical vagus nerve was dissected and separated from the aortic depressor nerve and the sympathetic trunk and was then cleaned from surrounding tissues in order to avoid any tissues between the cuff electrode and the nerve.

After finalization of in-vivo VNS experiments, which are described in the following section, animals were euthanized using pentobarbital (Release^®^, WDT, 300 mg/ml, 1.6 ml/kg bodyweight).

#### In-vivo setup and instrumentation

**Figure 2:**
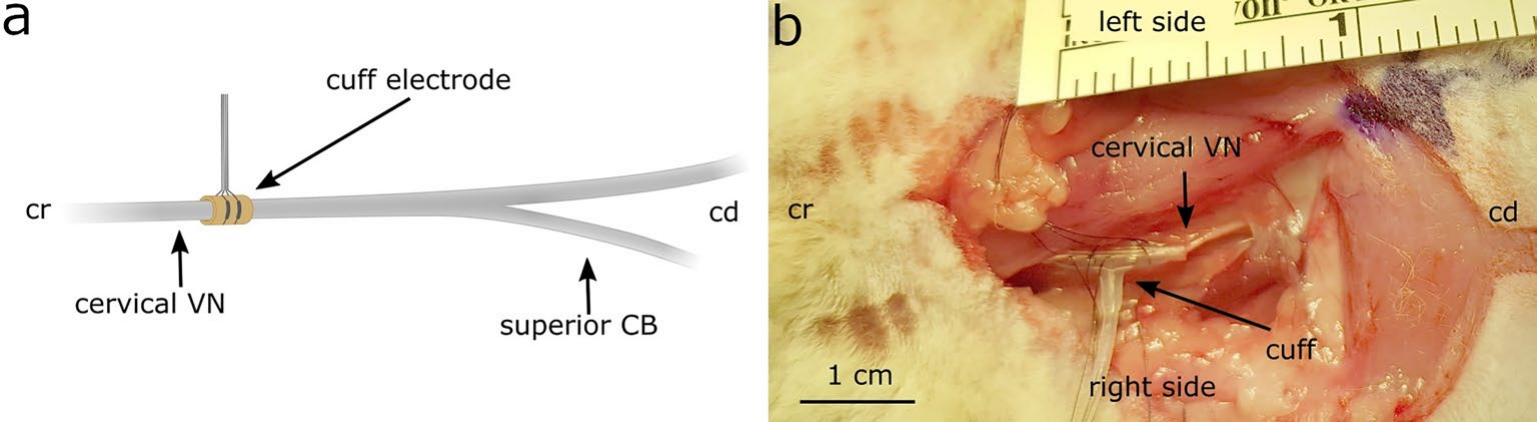
(**a**) Schematic of a cuff electrode wrapped around the cervical VN at mid-cervical level, approximately 4–5 cm cranial to the cardiac branching point of the superior cardiac branch. (**b**) Surgical window of a rabbit instrumented with the cuff electrode placed at the mid-cervical level. *Cr* cranial, *cd* caudal, *CB* cardiac branch, *VN* vagus nerve.

In-vivo VNS stimulation was performed at the mid-cervical level on the intact cervical VN**,** approximately 4–5 cm cranial to the branching point of the superior cardiac branch. The nerves were instrumented using a bipolar cuff-electrode of 0.75 mm diameter with contact spacing of 3 mm that was wrapped around the right cervical vagus nerve at the mid cervical level as shown in Fig. 2a,b. The leads of the electrode were connected to a linear isolated stimulator (STMISOLA, BioPac Systems). A standard 3-lead electrocardiogram (ECG) was acquired with the needle electrodes (MyoStim^®^ Bipolar Bifurcate) placed on the limbs. The ECG signal was pre-amplified, low-pass filtered at 1000 Hz and high-pass filtered at 1 Hz using a differential amplifier (Warner Electronics DP-304A). All data were digitized and recorded using a dSPACE MicroLabBox system and a custom-developed software.

#### Vagus nerve dissection ex-vivo

**Figure 3:**
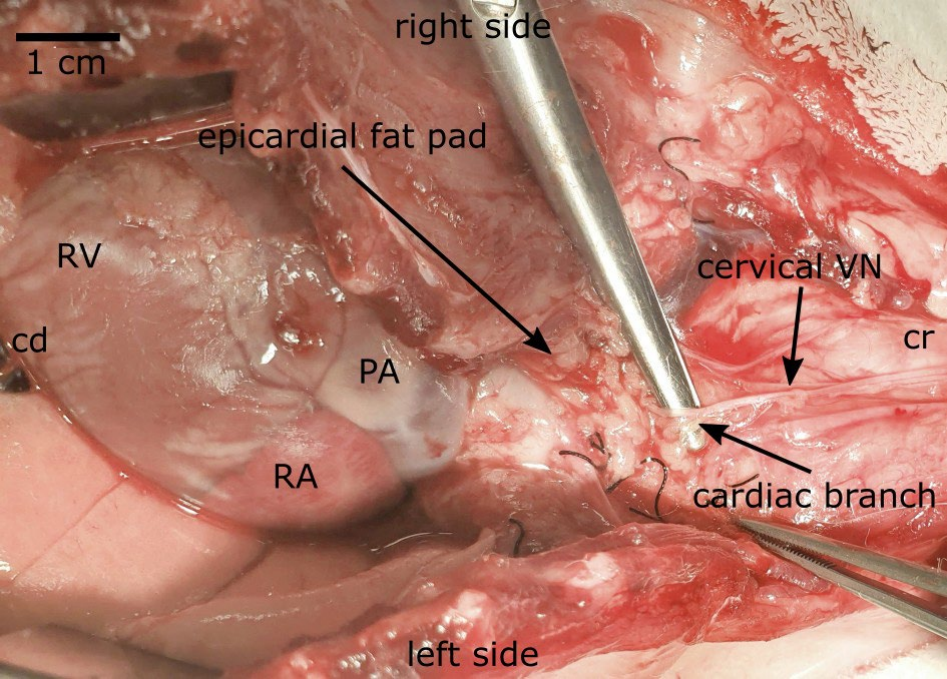
Surgical dissection of the vagus nerve (VN) for ex-vivo experiments including superior cardiac branch (CB). Here, the VN is shown from its caudal half of the cervical level to caudal to the superior CB. The heart is exposed after opening of the pericardium. *Cr* cranial, *cd* caudal, *PA* pulmonary artery, *RA* right atrium, *RV* right ventricle.

Vagus nerve dissection for ex-vivo experiments was performed as described previously^30,31^. Briefly, a median skin incision was made from the mandibles to the sternum and further extended to the sternal xiphoid. The right cervical muscles were identified, and the carotid sheath, containing the internal jugular vein, common carotid artery, the VN and sympathetic trunk (ST) was opened followed by blunt dissection of the VN from the nodose ganglion (NG) caudally to the thoracic aperture. The cardiac branching point, where the superior cardiac branch separated from the VN trunk, could be identified approximately between 0.5 and 2 cm cranial to the epicardial fat pad, approximately at the level of the thoracic aperture as shown in Fig. 3.

#### Isolation of the innervated isolated heart

The VN was completely dissected from the cervical level to the superior cardiac branch as described above. Next, the pectoral muscles were identified and cut to expose the subclavian vessels and the VN diving down under the muscles. Next, the thorax was opened by median sternotomy, the pericardium was opened and the right VN including the superior cardiac branch were further traced towards caudal until it dived into the epicardial fat pad as shown above in Fig. 3. Prior to excision of the heart, a bolus of heparin (1000 I.E./kg) was administered and the right VN was cut just above the nodose ganglion, followed by carefully separating the heart caudal from the thoracic arteries and cranial at level of the aortic arch. Care was taken to prevent the VN and the cardiac branch from mechanically rupture during surgery and heart excision. The heart was rapidly excised and immediately placed into ice-cold Krebs–Henseleit buffer.

#### Ex-vivo setup and instrumentation

Finally, the aorta was quickly cannulated, and the heart was mounted to the isolated heart system. Perfusion of the innervated isolated heart was performed under constant pressure (80 mmHg) in Langendorff mode with erythrocyte-enriched Krebs-Henseleit buffer in order to improve oxygenation of the cardiomyocytes. The nerve was kept moist throughout the experiment using isotonic sodium chloride (0.9%) to maintain vitality and excitability. Details on the setup of the isolated heart system and the chemical composition of the erythrocyte- enriched Krebs–Henseleit buffer are described in^32,33^.

**Figure 4:**
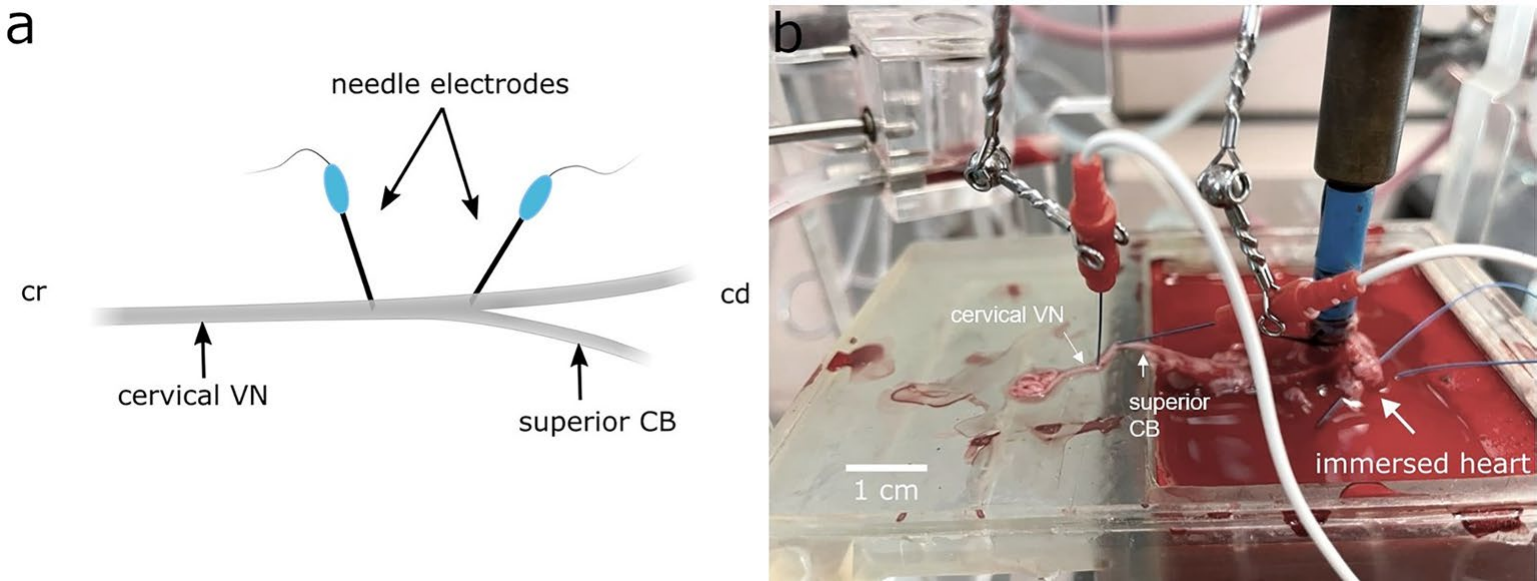
(**a**) Schematic showing the placement of needle electrodes. (**b**) Isolated innervated rabbit heart mounted to the working heart system. A pair of needle electrodes is placed close to the superior cardiac branch (cathode) and cranial to the cervical VN (anode). ECG leads were placed into the right atrium for heart rate detection. *cr* cranial, *cd* caudal, *CB* cardiac branch, *ECG* electrocardiogram, *VN* vagus nerve.

The right VN was instrumented ex-vivo using two concentric needle electrodes connected to a linear isolated stimulator (STMISOLA, BioPac Systems), same as used for in-vivo experiments, with the cathode placed approximately 2 to 5 mm cranial to the cardiac vagal branch (CB) and the anode placed 2–5 mm further cranial to the anode as shown in Fig.4a,b. The ex-vivo experiments were principally performed in preparation for heart rate control strategies using a trans-fascicular intraneural microelectrode (TIME) array. Therefore, intraneural stimulation for the cervical and cardiac vagus nerve was performed using a bipolar array of needle electrodes to mimic the stimulation using TIME electrodes.

In order to acquire ECG, two wire electrodes (MyoStim^®^ Bipolar Bifurcate) were inserted into the right atrium (Fig. 4b). Same as for in-vivo, the ECG signals were pre-amplified with a gain of 1000 using a differential amplifier low-pass filtered at 1000 Hz, high-pass filtered at 1 Hz (Warner Electronics DP-304A). Same as in-vivo, a dSPACE MicroLabBox system and a custom-developed software were used for digitalization and recording of data.

### Assessing the chronotropic effects of VNS in-vivo and ex-vivo

**Figure 5:**
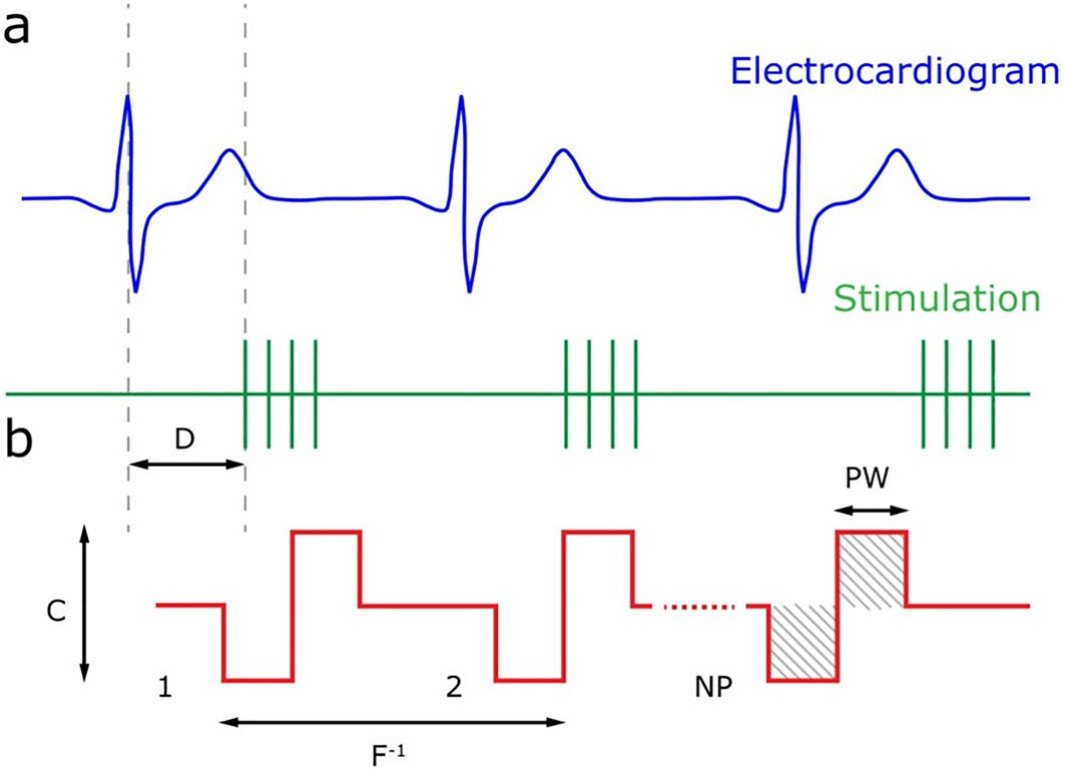
Schematic of the cardiac-synchronized stimulation. (**a**) Electrocardiogram (ECG) trace along with a stimulation burst with respect to the R-peak highlighted by the dashed lines in the ECG. (**b**) Detailed view of biphasic pulses applied per stimulation burst. *C* current amplitude, *PW* pulse width, *NP* number of pulses, *F* frequency, *D* delay between R-peak and stimulation onset.

In both setups, VNS was performed in synchronization with the cardiac cycle, where bursts of cathodic-anodic charge-balance rectangular stimulation pulses were applied in each cardiac cycle with respect to the R-peak of the ECG (Fig. 5a). Cardiac-synchronized stimulation may be defined by five main stimulation parameters: current amplitude (C), pulse width (PW), frequency (F), number of pulses (NP), and delay (D). The charge was calculated from the area under the stimulation signal curve (Fig. 5b).

The right VN was stimulated for 30 s, followed by 30 s stimulation pause. The general excitability of the VN was assessed through the ability to reduce the HR by 5 bpm. The sensitivity to stimulate was assessed as the charge needed to reduce the HR by 10 bpm.

In-vivo, VNS was performed in open-loop, testing different value combinations for the five main stimulation parameters in the ranges specified in Table 1.

**Table 1:** VNS stimulation parameters applied in-vivo and ex-vivo.

	In-vivo VNS	Ex-vivo VNS
Pulse width (µs)	50–200	500
Frequency (Hz)	10–40	30
Number of pulses	1–6	8
Delay (ms)	0–300	0

In the ex-vivo isolated heart, closed-loop stimulation was applied where the current amplitude was adjusted to reduce the error between the measured instantaneous HR and the set HR (reduction by 10 bpm from baseline) while all other stimulation parameters remained constant (Table 1).

The initial selection of the stimulation parameter ranges was determined based on a previous work by Ojeda et al., where they presented a novel closed-loop method that allowed an optimized adaption of the stimulation parameters to VNS applications^21^. Selection of parameter ranges was refined from pilot in-vivo experiments that were performed in anesthetized rabbits in our institution. Basically, the upper limits for current, pulse width, etc. were increased from the initial values in order to evoke greater cardiac effects. Further details on the stimulation strategies can be found in Haberbusch et al.^31,34^.

### Data processing and analysis

All data were processed using MATLAB R2022a (Mathworks, Natick, Massachusetts, United States) and GraphPad prism software (GraphPad Software, San Diego, CA, United States).

#### Comparison of baseline heart rates between in-vivo and ex-vivo condition

To ensure comparability of the HR in the ex-vivo isolated heart and in-vivo condition, the baseline HR prior to VNS was calculated for both preparations and is presented as mean and standard deviation. The mean baseline HR of the in-vivo group was calculated from recordings of HR over 5 min after surgical dissection and instrumentation of the VN when HR was settled at stable values. Similar to that, for the ex-vivo group, the HR was calculated as the mean HR recorded during surgical dissection of the VN and as 5 min mean after the heart was reperfused ex-vivo.

**Figure 6:**
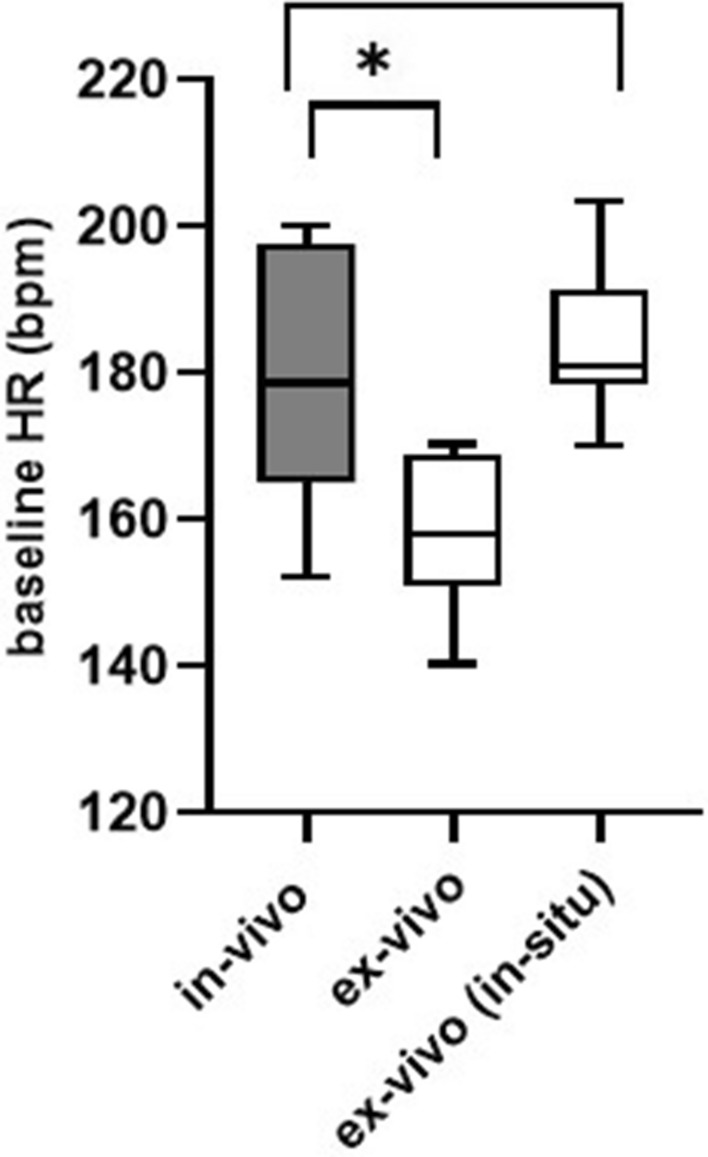
Comparison of the baseline heart rate in-vivo versus ex-vivo before and after heart explantation. Data are represented as mean and standard deviation (SD). The “*” symbol indicates statistical significance. Bpm: beats per minute. In-vivo: heart rate measurements after instrumentation in-vivo, in-situ: heart rate measurements prior to heart explantation in the ex-vivo animals, ex-vivo: after heart explantation in Langendorff mode, *HR* heart rate.

The baseline HR recorded in ex-vivo conditions, just prior to heart excision (in-situ) was determined for comparisons of the baseline HR in-vivo versus ex-vivo (in-situ) (Fig. 6) in order to ensure comparability of the two experimental setups.

#### Comparison of stimulation charges

In order to distinguish stimulation responses from physiological HR fluctuations, the physiological threshold charge was determined as the charge needed to reduce HR by 5 bpm. To compare the 10-bpm charges between ex-vivo and in-vivo condition, they were normalized with respect to the physiological threshold charge as listed in Table 2.

**Table 2:** Mean baseline heart rate and charges required to reach a reduction of heart rate by 5 bpm (physiological threshold charge) and 10 bpm (10-bpm-charge), respectively.

Parameter	In-vivo	Ex-vivo
Baseline HR (bpm)	181 ± 19	158 ± 11
Physiological threshold charge (µC)	549 ± 370	9 ± 6
10 bpm charge (µC)	918 ± 546	10 + 7
Normalized 10-bpm-charge (xPT)	1.78 ± 0.8	1.22 ± 0.1
Overshoot	3/5	2/6
Stimulation type	Open loop	Closed loop

10-bpm-charges were normalized to enable a comparison between both stimulation strategies in-vivo versus ex-vivo. Overshoot responses describe cases of short heart rate increases by 5 bpm after heart rate has returned to baseline after stimulation was turned off. xPT: physiological threshold charge. Data are given as mean and standard deviation.

For ex-vivo data, physiological threshold charges and charges needed for reducing the HR by 10 bpm were calculated from the last stimulation burst preceding the time point where the HR reached 5 bpm and 10 bpm reduction, respectively.

For the analysis of the in-vivo data, HR responses had to be pooled, because the stimulation parameters (Table 1 ) were not fixed but the parameters C, PW, NP, F and D as described in “Assessing the chronotropic effects of VNS in-vivo and ex-vivo”. were combined differently. For this purpose, the HR responses were binned in blocks of 5 bpm and the mean charge, and the threshold charge were calculated identically to the ex-vivo experiments.

#### Assessing waveform features of HR decrease after VNS ex-vivo and in-vivo

Curves representing the time-course of HR changes before, during, and after VNS were analyzed to observe peculiar dynamic features following stimulation, which were described as overshoots in this study.

#### Statistical analysis

Data analysis was performed using MATLAB R2022a (Mathworks, Natick, Massachusetts, United States) and GraphPad prism software (GraphPad Software, San Diego, CA, United States). The data were analyzed using descriptive statistics. Normal distribution of data was assessed by the Shapiro–Wilk test. An unpaired t-test was used to compare mean baseline HR and normalized charges between in-vivo and ex-vivo condition. An F-test was applied to compare variances of normalized 10-bpm charges between groups. Data is presented as mean ± standard deviation (SD). Results were deemed statistically significant for p < 0.05.

## Results

### Comparison of baseline heart rates between in-vivo and ex-vivo conditions

Baseline values obtained after preparation of the nerves and the heart [in-vivo: 181 ± 19 bpm, ex-vivo (in-situ) 182 ± 10 bpm] were similar in both preparations. After explantation, the baseline HR in the ex-vivo heart declined from 182 ± 10 bpm to 158 ± 11bpm. Differences in baseline HR between both setups are displayed in Fig. 6.

The difference between in-vivo and ex-vivo (in-situ) was not statistically significant (unpaired t-test, p = 0.21, Fig. 6), whereas the difference between mean baseline values in-vivo and ex-vivo post-explantation groups was statistically significant (unpaired t-test, p = 0.03, Fig. 6).

### Stimulation charges needed for different levels of chronotropic responses

The charges needed to reduce the HR by 5 bpm were 549 ± 370 µC in-vivo and 9 ± 6 µC ex-vivo. To reach a 10-bpm HR reduction, the respective charges were normalized to the physiological threshold charge, was 1.78 ± 0.8 in-vivo and 1.22 ± 0.1 ex-vivo (Table 2).

**Figure 7:**
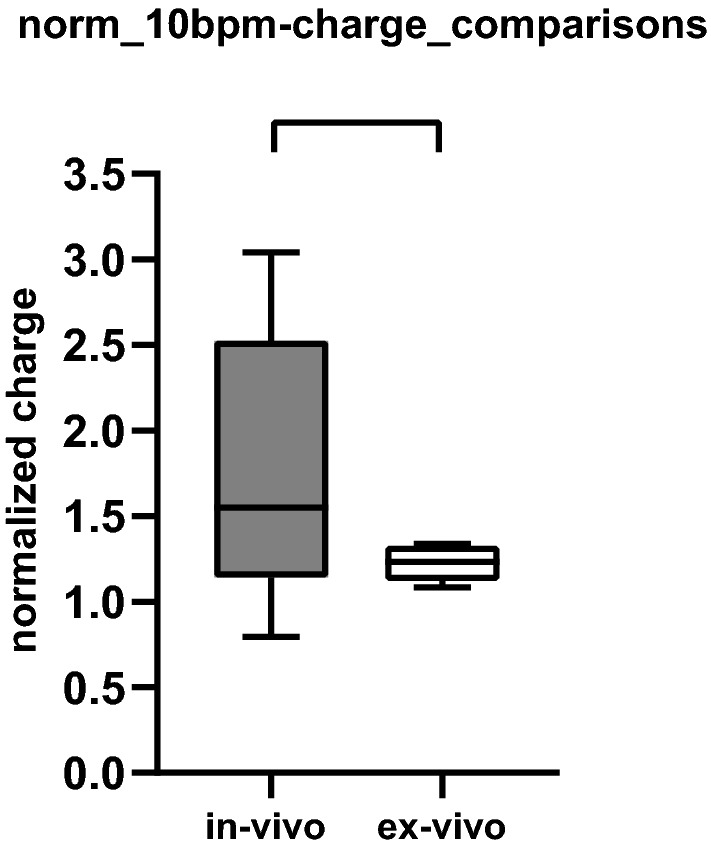
Comparison of the normalized charges required to reduce heart rate by 10 bpm ex-vivo versus in-vivo. Data represent the mean values from five experiments in-vivo ex-vivo, and six experiments ex-vivo. Data are presented as mean and standard deviation (SD).

Next, the differences between mean normalized 10-bpm charges in-vivo and ex-vivo (Fig. 7) were compared, which was 0.6 ± 0.3 xPT, being not statistically significant (two-tailed t-test, p = 0.14) In contrast, the variance of normalized 10-bpm charges were significantly different in-vivo and ex-vivo condition (F-test, *p* = 0.0005), suggesting that greater variations of in-vivo than ex-vivo in order to achieve a 10-bpm HR reduction.

### Overshoots observed after VNS ex-vivo and in-vivo

**Figure 8:**
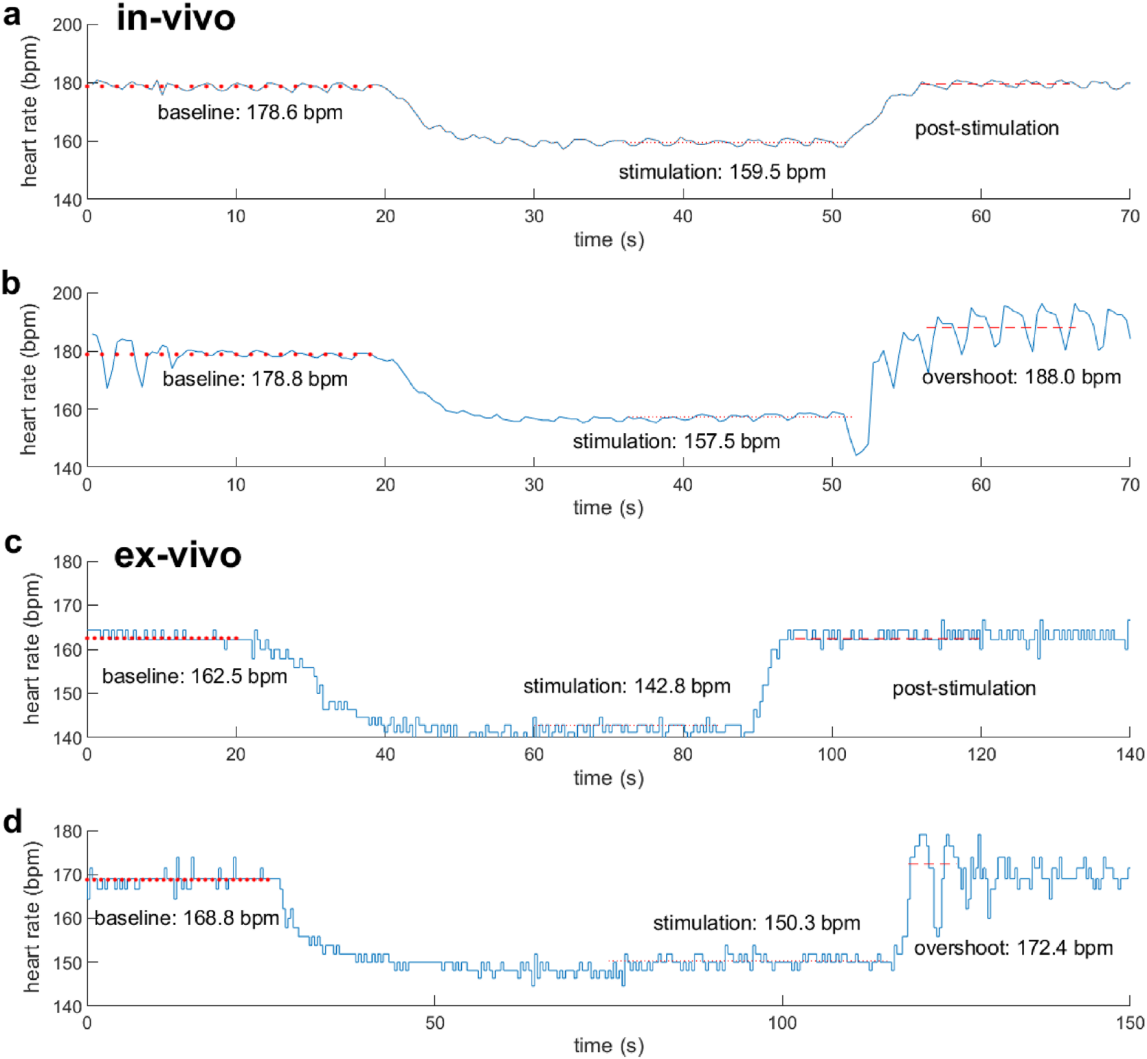
Representative ECG recordings for in-vivo (**a,b**) and ex-vivo VNS (**c,d**), each represented without and with overshoot. Curves a and c represent the ECG curves without overshoot in-vivo (**a**) and ex-vivo (**c**), respectively (“post stimulation”). Curves (**b,d**) represent the ECG curves with overshoot in-vivo (**b**) and ex-vivo (**d**), respectively after stimulation was turned off. The onset of stimulation is demonstrated by the decrease of heart rate in this ECG, whereas the stop of the stimulation is highlighted increasing heart rate. Overshoots are defined as an immediate increase of heart rate by at least 5 bpm above baseline after stimulation was turned off, followed by a return of heart rate back to the actual baseline.

Overshoots were observed in several experiments in-vivo and even ex-vivo. In both setups, an overshoot was detectable as a short rise of HR about 5–10 bpm above baseline, after VNS was turned off, followed by a return to baseline. Out of all experiments, three overshoots were observed in 3/5 experiments in-vivo and two overshoots were observed in 2/6 experiments ex-vivo (Table 2) as also exemplarily shown in Fig. 8. Here, the HR curves before, during and after VNS for the in-vivo (Fig. 8a,b) and ex-vivo conditions **(**Fig. 8c,d**)** are presented. Figure 8a,c represent the HR curves without overshoot in-vivo and ex-vivo, respectively (“post stimulation”). Figure 8b,d represent the HR curves with overshoot in-vivo and ex-vivo, respectively. In Fig. 8a an exemplary HR response to open-loop VNS in the in-vivo experiment shows a HR reduction by approximately 19.1 bpm from 178.6 bpm baseline HR to 159.5 bpm after stimulation. There was no overshoot present. Figure 8b shows the HR change in response to open-loop VNS with overshoot. The HR was reduced by 21.3 bpm, which is similar to the stimulation response presented in Fig. 8a. However, interestingly we can see an overshoot of the HR after stimulation was turned off, represented by a pronounced increase of the HR to 9.2 bpm above baseline before it eventually settled to the baseline level again.

Figure 8c,d represents an exemplary HR response for closed-loop VNS in the ex-vivo isolated heart. Here, the HR declined (Fig. 8c) from 162.5 to 142.8 bpm by 19.7 bpm without overshoot. Figure 8d shows the HR change in response to closed-loop VNS with overshoot. The HR was reduced by 18.5 bpm from 168.8 to 150.3 bpm, which is quite similar to the stimulation response presented in Fig. 8c. In contrast, this HR curve (Fig. 8d) shows an overshoot of the HR after stimulation was turned off, as presented by a conspicuous increase of the HR to 172.4 bpm before it returns to the baseline level again.

## Discussion

Vagus nerve stimulation (VNS) has proven to be an organ-protective and potential alternative therapeutic approach to treat various pathological conditions by restoration of the autonomic balance, such as atrial fibrillation^13^, ventricular arrhytmias^15^, persistent tachycardia^35^, and heart failure^2,36,37^. However, previous studies have also shown that in-vivo VNS is still mainly performed at the cervical level of the VN, which has shown to often provoke unwanted off-target effects, and consequently limits the therapeutic efficacy of those applications^30,38,39^. In cardiac medicine, in-vivo VNS has shown to have strong impact on cardiac activity, such as by reduction of HR and atrioventricular conduction^40^. The decrease of HR after VNS is also accompanied by activation of antagonistic hemodynamic feedback loops and inter-individual variations, which further complicates the investigations of specific effects of VNS strategies in-vivo^14,41,42^. As an alternative attempt to account for this problem, the research group of Ng., Brack et al. have previously presented a model of in-situ innervated isolated rabbit heart, in which the heart was separated from the circulation but was still left in-situ. Both autonomic nerves were dissected and stimulated in order to investigate the direct stimulation effects on cardiac activity and atrioventricular conduction^26,43–45^. However, both approaches of VNS, in-vivo and in-situ, could not yet provide a model to study target-specific effects of VNS, such as more selective stimulation of the cardiac VN in a fully ex-vivo isolated innervated heart model.

In this study, a novel experimental setup of a fully ex-vivo isolated innervated rabbit heart with vagal innervation is presented that allows for investigations of acute cardiac effects of VNS. Our ex-vivo model delivered reproducible results in n = 6 isolated innervated rabbit hearts, therefore potentially providing an experimental setup for VNS without interventions of physiological reflexes and inter-individual variations.

This present study, to the best of our knowledge, is the first approach that investigated the feasibility and viability of stimulating a fully ex-vivo innervated isolated heart by comparing applied stimulation charges in this ex-vivo model to well-studied models of in-vivo VNS^21,22,46,47^. The in-vivo experiments were performed using a cuff electrode as this is a commonly used non-invasive approach for cervical VNS. The ex-vivo experiments, in contrast, were performed in preparation for heart rate control strategies using a trans-fascicular intraneural microelectrode (TIME) array. Therefore, ex-vivo stimulation was performed as an intraneuronal stimulation approach using a bipolar array of needle electrodes mimicking the stimulation with two TIME contacts, which serves the purpose to reduce off-target stimulation and increase the cardiac selectivity. For the ex-vivo stimulation of the VN, the stimulation site was moved closer to the heart and stimulated the VN just proximal to the superior cardiac branch using needle electrodes, which is also described in Berthoud et al.^48^ and Haberbusch et al.^49^. The fact that this intraneural approach is more invasive compared to the approach of cervical VNS using cuff electrodes, however, limits this kind of study to acute investigations. Nevertheless, this approach of intraneural stimulation provides a potential improvement of acute experiments for selective cardiac VNS with recruitment of an increased number of cardiac and less off-target fibers^30,50–52^.

The evaluation of the viability and comparability of this ex-vivo approach was performed by comparing this ex-vivo model to an already well-established in-vivo experimental setup. Firstly, the baseline HR obtained were investigated in-vivo and ex-vivo in order to ensure that comparable physiological conditions were given before VNS was performed. Therefore, the baseline HR was compared between the in-vivo group versus the ex-vivo group before explantation (ex-vivo “in-situ”, Fig. 6) and after heart explantation (“ex-vivo”, Fig. 6). It turned out that the mean baseline HR in-vivo and ex-vivo (in-situ) was almost the same in all animals. In contrast, a significant decrease of HR was observed ex-vivo after heart explantation versus in-vivo (from 182 ± 10 bpm to 158 ± 11 bpm (p = 0.014)). Comparing the findings of this study to literature, it turned out that our recorded baseline HR ex-vivo were similar to those reported by Brack et al., who measured baseline HR in Langendorff-perfused rabbit hearts about 146 ± 2^10^ and 153 ± 4 bpm^25^. A possible reason for the abrupt decrease of baseline HR ex-vivo are most likely caused by multiple factors. One possible explanation is that, from a physiological perspective, the transfer of the heart and the de-afferentiated nerve to ex-vivo conditions cause a sudden shift in the cardiac metabolism and in the electrophysiology of the VN. The activity of sinoatrial node cells, for instance, slows down when changes of temperature occur in their environment, which is the case when the heart was briefly cooled down after explantation in order reduce the cardiac metabolism and the speed of degradation^53^. Additionally, during the short time frame between heart explantation and reperfusion of the isolated innervated heart, one cannot exclude the occurrence of reperfusion injuries and short periods of ischemic conditions.

Secondly, the charges that were required to reduce HR by 5 bpm (physiological threshold charge) and 10 bpm were compared in-vivo versus ex-vivo. Data has shown that the absolute charges applied for VNS greatly varied between both setups. In-vivo, a tendence towards higher absolute charges was observed to elicit a chronotropic response compared to ex-vivo and the inter-individual differences were also greater in-vivo compared to ex-vivo. Therefore, in order to reach a better comparability of the data by reducing the differences between both data sets, the 10-bpm charges were normalized to the physiological threshold charge. The normalized data have revealed a greater inter-individual variation of charges in-vivo versus ex-vivo but still delivered satisfying reproducibility and controllability of the stimulation results. In contrast, the data of absolute charges demonstrated the greater inter-individual differences of applied charges in-vivo versus ex-vivo, which possibly emphasizes the impact of the type of electrodes used for stimulation on the one hand and individual reactions in-vivo to anesthesia, circulating catecholamines, and the presence of autonomic feedback loops on the other hand^17,54,55^.

Lastly, the number of observations of HR overshoot responses after termination of VNS was counted in the data sets of in-vivo and ex-vivo experiments. There are various reasons for the occurrence of such events, all being related to autonomic cardiac control. However, since we observed these events not only in-vivo but also ex-vivo, we compared the occurrence of these events in both experimental conditions. Overshoot events were observed in 3/5 in-vivo and 2/6 ex-vivo experiments (Fig. 8). Interestingly, overshoots were also found in the ex-vivo recordings, even though the heart was isolated from the neuronal control centers and the circulating catecholamines in the hemodynamic system. Previous studies have stated that the autonomic control of the heart is regulated at three stages, namely by the central, intrathoracic, and intracardiac level and that all three centers communicate via afferent and efferent directions with each other^56–58^. The intracardiac regulation is composed of ganglionated sympathetic and parasympathetic plexuses, which are embedded in the epicardial fat pad forming an interconnected neuronal network that integrates neural signals between intracardiac ganglia and higher cardiac regulation centers^59,60^. A former study by Hanna et al. reported about a similar observation, which was referred to as “post-vagal tachycardia”^61^. This observation was explained by the fact, firstly, that cholinergic and catecholaminergic neurons are anatomically clustered in ganglia and interconnected near the sinoatrial node. Secondly, both groups of cells are involved in this biphasic response to initial reduction and subsequent increase of HR during stimulation of the intracardiac nerve cells. Hence, we hypothesize that the occurrence of overshoots in our ex-vivo study might also be explained by the intracardiac circuits and crosstalk between intracardial sympathetic and parasympathetic ganglia as the HR decreases.

One aim of this study, besides selective cardiac VNS ex-vivo, was the surgical development of this model as well as its establishment by means of a proof-of-concept, which was achieved with a HR reduction of at least 5 to 10 bpm. In particular, the cofounding factors described in this study (surgery, invasiveness of the intraneural electrodes, stimulation protocols) are important to be taken into consideration. Therefore, a series of pilot experiments was necessary in which both, open-loop and closed-loop stimulation approaches, were investigated. From a series of 25 rabbit hearts, six hearts could be used for a complete closed-loop VNS protocol. Out of the remaining 19 hearts, we could provoke stimulation responses in 16 hearts, of which 8 hearts were used to establish the setup and instrumentation. This work completes a previous work by our group, demonstrating the approach of closed-loop VNS in this isolated-heart setup^31^.

In sum, this study highlights the challenges and advantages of an ex-vivo experimental setup and provides a novel approach towards advanced experimental setups for cardiac neuromodulation under defined experimental conditions. It further helps to gain a deeper understanding of specific questions with respect to effects of VNS on electrophysiology and cardiac physiology^31,41^.

### Limitations of the study

The main limitation of this study is that we used two different types of electrodes in the ex-vivo and in-vivo setup. While this compromises a direct comparability of the results, normalizing the 10-bpm charges to the physiological threshold charge still allows the two settings to be compared. The use of two different types of electrodes and the presence in-vivo of physiological factors absent in the ex-vivo setup are most likely causing the differences in stimulation thresholds in the two setups. The quantification of the relative importance of these two effects were not performed systematically apart from the proposed normalization strategy. Also, an evaluation of VNS stimulation in the ex-vivo and in-vivo setups in terms of ECG derived parameters (e.g. heart rate variability, arrhythmia) would be also advisable, but could not be performed in the current setting. Therefore, follow-up research and experimentation efforts are ongoing to the use of intraneural electrodes also in-vivo for direct comparability and also to extend the ex-vivo system to the so-called working mode (closed-loop circulation) in which an extensive full characterization in terms of chronotropic, dromotropic and inotropic responses to VNS can be performed.

Furthermore, we expect to improve this model to systematically provoke greater ranges in HR reduction that are comparable to current therapeutic applications of VNS.

## Conclusion

To conclude, in this study we presented a novel ex-vivo innervated isolated heart model that allows for investigation of direct cardiac effects of VNS in absence of uncontrollable cofounding factors, as present under in-vivo conditions. We could stimulate the VN from the cervical level to the superior cardiac branch and consistently achieved physiological heart responses for several hours. Lastly, we showed that the normalized charges for HR reduction were similar in this ex-vivo preparation compared to the in-vivo setup. Overall, this study provides promising results that pave the way for further investigations in this field.

## Data Availability

The data that support the findings of this study are available from the corresponding author upon reasonable request.
